# An investigation into epidemiological situations of COVID-19 with fuzzy K-means and K-prototype clustering methods

**DOI:** 10.1038/s41598-023-33214-y

**Published:** 2023-04-17

**Authors:** Ozge Pasin, Senem Gonenc

**Affiliations:** 1 Biostatistics Department, Faculty of Medicine, Bezmialem University, Fatih, Istanbul, Turkey; 2 Statistics Department, Faculty of Science, Ataturk University, Erzurum, Turkey

**Keywords:** Immunology, Medical research, Mathematics and computing

## Abstract

The ten countries with the highest population during the pandemic were analyzed for clustering based on the quantitative numbers of COVID-19 and policy plans. The Fuzzy K-Means (FKM) and K-prototype algorithms were used for clustering, and various performance indices such as Partition Coefficient (PC), Partition Entropy (PE), Xie-Beni (XB), and Silhouette Fuzzy (SIL.F) were used for evaluating the clusters. The analysis included variables such as confirmed cases, tests, vaccines, school and workplace closures, event cancellations, gathering restrictions, transport closures, stay-at-home restrictions, international movement restrictions, testing policies, facial coverings, and vaccination policy statuses. PC, PE, XB, and SIL.F indices were used to analyze the performance indices of the clusters. The Elbow method was used to analyze the performance evaluations for the K-prototype. The K-prototype algorithm's performance evaluations were analyzed using the Elbow method, and the optimum number of clusters for both methods was found to be two. The first cluster included Brazil, Mexico, Nigeria, Bangladesh, US, Indonesia, Russia, and Pakistan, while the second cluster comprised India and China. The analysis also examined the relationship between population and confirmed tests and vaccines, and standardization was made for the country with the largest population for significant correlations. The results showed that the FKM method was superior to the K-prototype method in terms of clustering. In conclusion, it is crucial to accurately evaluate COVID-19 data for countries and develop appropriate policies. The clustering analysis using the FKM and K-prototype algorithms provides valuable insights into identifying groups of countries with similar COVID-19 data and policy plans.

## Introduction

COVID-19, a disease caused by the novel coronavirus, was first reported in December 2019 in the city of Wuhan, located in Hubei Province in China. The virus quickly spread to other countries, with cases being reported in January 2020 and the pandemic rapidly escalating into a global health crisis. The World Health Organization (WHO) provides daily updates on the number of COVID-19 cases and deaths worldwide. On March 11, 2020, the WHO declared COVID-19 a global pandemic due to the widespread and rapid transmission of the virus across different countries and continents. According to the data published on July 22, 2022, the number of cases in the world was 573,032,061, while the number of deaths was 6,398,987^1^. As the number of COVID-19 cases continues to rise, there has been a gradual increase in the number of people who are getting vaccinated. This is being done to slow the spread of the virus and to protect individuals, particularly those who are at higher risk of severe illness or death from COVID-19. Numerous studies are being conducted in this field because this growth has become a problem on a global scale. In order to control the spread of COVID-19, different countries have implemented various policies and measures. Some countries have closed their borders or restricted international travel, while others have closed schools or imposed restrictions on certain activities, such as large gatherings or indoor dining. These policies are being put in place to reduce the number of infections and to slow the spread of the virus, with the ultimate goal of protecting public health and reducing the burden on healthcare systems. The specific policies implemented vary by country and are based on a variety of factors, including the current state of the pandemic, the local healthcare capacity, and the economic and social impact of the measures.

The scientific literature has described several mathematical models that have been used to study and understand the spread of COVID-19. However, it is uncommon to find research that use advanced clustering approaches to assess both policies and COVID-19 statistics regarding the pandemic. The aim of this study is to cluster countries based on their characteristics, including the policies implemented in response to COVID-19, as well as the numbers of confirmed cases, tests conducted, and vaccines administered. The clustering approach can help to identify patterns and trends in the data, and may provide insights into the effectiveness of different policy approaches in controlling the spread of the virus. By understanding these patterns, policymakers and public health officials can make more informed decisions about how to respond to the pandemic, and allocate resources more effectively to manage the ongoing public health crisis. In order to cluster countries based on their characteristics related to COVID-19, advanced statistical methods such as Fuzzy K-means (FKM) and K-prototype methods were used. Many studies have evaluated the epidemiological factors and behavioral patterns related to COVID-19. In the clinical sense, studies have been conducted that evaluate many different aspects such as age, gender, health system infrastructures, social cohesion, etc. Although many studies have grouped countries based on the number of COVID-19 cases and deaths, there is a lack of research that evaluates these changes over a longer period of time, and considers the policies implemented by countries in conjunction with detailed features using advanced clustering methods. In this sense, it is thought that this study will be a pioneering study in the literature. Therefore, it is thought that a study that combines these aspects would be pioneering in the literature. By analyzing COVID-19 data over a longer time period and considering a range of factors, such as policy implementation, demographic and health characteristics, and socioeconomic factors, an advanced clustering method can identify meaningful patterns and trends in the data. Such a study can help policymakers and public health officials to better understand how different countries have responded to the pandemic, and identify successful strategies that can be applied in other contexts. In addition, this type of analysis can help to identify gaps and areas where more resources and attention are needed to control the spread of the virus and mitigate its impact on vulnerable populations. Overall, a pioneering study that uses advanced clustering methods to evaluate COVID-19 data can contribute to the ongoing efforts to manage this global health crisis.

### Clustering with fuzzy K-means

Clustering methods are techniques that enable data to be gathered into distinct clusters based on their similarity to each other with respect to variables. Clustering methods are utilized in diagnostic systems, in the field of bioinformatics, and in various stages and areas such as decision-making for patient treatment. In clustering methods, the first step is to determine the primary criterion for the clustering process. Subsequent steps include variable selection, standardization of variable values, selection of a clustering method, determination of the number of clusters, and identification of empirical types, respectively. The literature includes a wide range of clustering algorithms. There are many clustering algorithms in the literature depending on the application conditions and data structures. In general, clustering methods are classified into hard clustering and soft clustering methods, based on the conditions or constraints of membership to the cluster^2^. The fuzzy clustering method is one of the clustering algorithms that performs soft clustering. When utilizing fuzzy clustering techniques, data units may be assigned to two or more clusters based on the various membership degrees they exhibit. Due to this, and the fact that data units on the boundaries of clusters are not required to belong to a specific cluster, fuzzy clustering offers a greater and more organic potential for clustering than hard clustering. FKM, a clustering technique among these fuzzy algorithms, is employed in the literature for various purposes and in many different disciplines. FKM, which was initially developed by Bezdek in 1981, is the most widely recognized algorithm among fuzzy clustering methods^3^. According to Yang and Sinaga, this method has been “widely extended and applied in various real-world problems, such as pattern recognition, image segmentation, medical diagnostic, economics, cell formation, gene expression, and data mining^4^”. This algorithm's goal is to divide *n* observations into *k* clusters in the most optimal way possible using fuzzy partition. In order to find the best fuzzy partition, the following function is minimized^5^.

$${min}_{U,H}{J}_{FKM}=\sum_{i=1}^{n}\sum_{g=1}^{k}{u}_{gi}^{m}{d}^{2}({x}_{i},{v}_{g}), {u}_{gi}\epsilon \left[\mathrm{0,1}\right], {\sum }_{g=1}^{k}{u}_{gi}=1$$

The term *d(*${x}_{i},{v}_{g}$*)* in the above function is the Euclidean distance. Cluster centers, called Prototypes in the method, represent each cluster. Euclidean distance between observations and cluster centers is preferred as a measure of distance^6^.${u}_{gi}$ is the generic element of the membership value of the *U* matrix and it takes values between 0 and 1. It shows the cluster memberships.${v}_{g}$: prototype (centres) vector for cluster *g*.${x}_{i}$: feature vector for data point *i*.${u}_{gi}$: fuzzy membership degree of data point *i* to the cluster *g.m*: weighting exponent for fuzziness.*k*: an integer defining the number of clusters to be used in clustering.

In the FKM algorithm, the observations are assigned to higher order clusters, but there is also a varying degree of membership of observations to different clusters. This algorithm involves a structure that is updated iteratively with randomly assigned values. Through this iterative process, the FKM algorithm locates the cluster centers in the appropriate positions within the dataset by the end of the algorithm. The most notable feature of the FKM algorithm is the membership matrix of the dataset, which is generated along with the clusters formed as a result of clustering. This matrix enables various inferences to be made by clarifying the uncertain situations that exist between the resulting clusters and each observation.

Different index coefficients are utilized in the method to evaluate the performance of clusters. There are many indices in the literature for evaluating the quality of clusters. In this study, the fuzzy cluster validity indices Xie-Beni index (XB), Silhouette Fuzzy (SIL.F), partition coefficient (PC), partition entropy (PE), and PC were utilized to evaluate the quality of clusters.

### Partition Coefficient (PC)

The partition coefficient (PC) was developed by Bezdek in 1974. The coefficient is calculated solely from fuzzy membership values. It is computationally efficient and straightforward. Additionally, for datasets containing round or spherical clusters, it is reported to be equally effective as the PE and XB indices described below, and is reported to be better than XB when the number of clusters selected is larger. ^7,8^.

$$\mathrm{PC}\hspace{0.17em}=\hspace{0.17em}{V}_{PC}\left(U\right)=\frac{1}{n}(\sum_{g=1}^{k}\sum_{i=1}^{n}{u}_{gi}^{m})$$

The formula in the equation ensures that the ${V}_{PC}$ values are in the range of $\left[1/k,1\right].$ When the index values become closer to 1, hard clusters are identified, while index values that are close to the lower limit indicate that the clusters are fuzzy. If there is a value such as *1/k*, the membership degrees of all members of the cluster are equal $\left({u}_{gi}=1/k\right)$. This implies that either there is no discernible clustering trend in the dataset or that the algorithm used has not been successful^9^. Since the purpose of clustering is to make clusters that are strictly separated from each other, the $max\left({V}_{PC}\right)$ value gives the best clustering.

### Partition Entropy (PE)

PE is another fuzzy validity index proposed by Bezdek in 1974, and it is formulated as shown in the equation below^7^.

$${\mathrm{PE}\hspace{0.17em}=\hspace{0.17em}V}_{PE}\left(U\right)=\frac{1}{n}(\sum_{g=1}^{k}\sum_{i=1}^{n}{u}_{gi}{log}_{a}({u}_{gi}))$$

In the formula, $a$ denotes a logarithm base. ${V}_{PE}$ is obtained in the range of $\left[0, {log}_{a}k\right]$. Contrary to ${V}_{PC}$ index values, when ${V}_{PE}$ values are small, that is, close to 0, well-separated clusters are obtained. Cluster structures begin to become fuzzy as the data approaches the upper bound. A ${V}_{PE}$ index value equal to ${log}_{a}k$ indicates no clustering tendency in the data structure or a complete failure of the algorithm used. Therefore, $min\left({V}_{PE}\right)$ is the index value that gives the best clustering ^3^.

### Xie–Beni (XB)

Xie and Beni (1991) developed the Xie–Beni Index (XB; Xie–Beni Index), which considers cluster compactness in the denominator and cluster segregation in the denominator^10^.

XB = $${V}_{XB}\left(U;V;X\right)=\frac{\sum_{g=1}^{k}\sum_{i=1}^{n}{u}_{gi}^{m}{\Vert {x}_{i}-{v}_{g}\Vert }^{2}}{n({min}_{1\le g,j\le k;g\ne j}\left\{{\Vert {v}_{g}-{v}_{j}\Vert }^{2}\right\})}$$

The numerator term of the equation measures the compactness of the fuzzy clustering by taking into account the distances of the units in a cluster from their cluster centers, while the denominator term considers the distances between the cluster centers and the clusters’ segregation power. Small values of the XB index indicate that compact, well-separated clusters were obtained^10^.

### Silhouette Fuzzy (SIL.F)

An extension proposed for evaluating fuzzy clustering methods incorporates silhouettes and fuzzy values into an average silhouette-based index, which is derived from the defuzzified partition^11^. The authors recommend calculating a weighted mean, where each silhouette is given a weight based on the difference between the two highest fuzzy membership values of the corresponding point.

$${\mathrm{SIL}.\mathrm{F}=V}_{FSil}\left(U,V,X\right)=\frac{{\sum_{i=1}^{n}({u}_{gi-{{u}_{gi}}^{^{\prime}}})}^{\alpha }{s}_{i}(k)}{{\sum_{i=1}^{n}({u}_{gi-{{u}_{gi}}^{^{\prime}}})}^{\alpha }}, {s}_{i}\left(k\right)=\frac{{b}_{i}-{a}_{i}}{\mathrm{max}({b}_{i},{a}_{i})}$$

${a}_{i}$ is the average dissimilarity between a data point and all data units belonging to the same cluster.${b}_{i},$ is the smallest mean dissimilarity for any of the other sets where a data point is not a member. ${u}_{gi}$ ve ${{u}_{gi}}^{^{\prime}}$ are the first and second largest elements of the membership matrix *i*. $\alpha$ is a weighing coefficient and is generally used as equal to 1. The optimal number of clusters *k* is such that the index takes the maximum value.

### Clustering with K-prototype

In the K-prototype method, which is used for mixed data, clusters are created by minimizing the total within-cluster distance using the mean for numerical variables and the mode for categorical variables. Initially, a set of random prototypes is created in the method. The K-prototype method is used for clustering mixed data and aims to create clusters that minimize the total within-cluster distance by using means for numeric variables and modes for categorical variables. In this method, a set of random prototypes is first generated, and then the closest prototype is chosen based on distance. The average and mode values of the variables are updated at each stage, the prototypes are updated, and these processes continue until the total within-cluster distance is minimized.When evaluating the performance of the clusters, the Elbow method is commonly used. This method suggests that the cluster number with the greatest amount of bending should be chosen as the ideal cluster. The objective function for the K-prototype is obtained using the following formula^12^.

$$E=\sum_{i=1}^{n}\sum_{g=1}^{k}{u}_{gi}d\left({x}_{i},{v}_{g}\right)$$

${x}_{i}$ are the observations in the dataset, ${v}_{g}$ are the cluster prototypes of observations. ${u}_{gi}$ are the elements of the *U* binary partition matrix. The distance measure used in the K-prototype method is obtained with the following function.

$$d\left({x}_{i},{v}_{g}\right)=\sum_{m=1}^{q}{\left({x}_{i}^{m}-{v}_{g}^{m}\right)}^{2}+\lambda \sum_{m=q+1}^{p}\delta \left({x}_{i}^{m},{v}_{g}^{m}\right)$$

The variable *m* is an index containing the numeric (*q*) and qualitative (*p*) variables. *d()* corresponds to the weighted sum of the Euclidean distance between two points in metric space and the simple matching distance for categorical variables. The λ parameter provides the balance between these two terms. For λ = 0, the effect of categorical variables is eliminated and only numerical variables are considered, as in traditional k-means clustering^13^.

## Material and methods

In this study, data related to COVID-19 and national policy plans from the ten most populated countries during the pandemic (Russia, China, Brazil, the United States, India, Pakistan, Indonesia, Bangladesh, Mexico, and Nigeria) were compared to determine whether they could be grouped together based on similarities. The data was obtained from the COVID-19 package in the R program. The clustering structures of the countries were examined by analyzing their confirmed cases, tests, vaccines, school closures, workplace closures, event cancellations, gathering restrictions, transport closures, stay-at-home restrictions, international movement restrictions, testing policy, facial coverings, and vaccination policy. The category definitions of the variables are provided in Table 1.

**Table 1:** Policy situations implemented by countries during the COVID-19 process.

Variable	Category
School closures	0—No measures 1—Recommend closing or all schools open with alterations resulting in significant differences compared to non-COVID-19 operations 2—Require closing (only some levels or categories, eg just high school, or just public schools) 3—Require closing all levels
Workplace closures	0—No measures 1—Recommend closing (or recommend work from home) or all businesses open with alterations resulting in significant differences compared to non-COVID-19 operation 2—Require closing (or work from home) for some sectors or categories of workers 3—Require closing (or work from home) for all-but-essential workplaces (eg., grocery stores, doctors)
Cancelling events	0—No measures 1—Recommend canceling 2—Require canceling
Gathering restrictions	0—No restrictions 1—Restrictions on very large gatherings (the limit is above 1000 people) 2—Restrictions on gatherings between 101–1000 people 3—Restrictions on gatherings between 11–100 people 4—Restrictions on gatherings of 10 people or less
Transport closures	0—No measures 1—Recommend closing (or significantly reducing volume/route/means of transport available) 2—Require closing (or prohibiting most citizens from using it)
Stay home restrictions	0—No measures 1—Recommend not leaving the house 2—Require not leaving the house with exceptions for daily exercise, grocery shopping, and ‘essential’ trips 3—Require not leaving the house with minimal exceptions (eg., allowed to leave once a week, or only one person can leave at a time, etc.)
Internal movement restrictions	0—No measures 1—Recommend not to travel between regions/cities 2—Internal movement restrictions in place
International Movement Restrictions	1—Screening arrivals 2—Quarantine arrivals from some or all regions 3—Ban arrivals from some regions 4—Ban on all regions or total border closure
Testing policy	0—No testing policy 1—Only those who both (a) have symptoms AND (b) meet specific criteria (eg., key workers, admitted to hospital, came into contact with a known case, returned from overseas) 2—Testing of anyone showing COVID-19 symptoms 3—Open public testing (eg., “drive through” testing available to asymptomatic people)
Facial coverings	1—Recommended 2—Required in some specified shared/public spaces outside the home with other people present, or in some situations when social distancing not possible 3—Required in all shared/public spaces outside the home with other people present or in all situations when social distancing is not possible 4—Required outside the home at all times regardless of location or presence of other people
Vaccination policy	0—No availability 1—Availability for ONE of the following: key workers/ clinically vulnerable groups (non-elderly)/elderly groups 2—Availability for TWO of the following: key workers/ clinically vulnerable groups (non-elderly)/elderly groups 3—Availability for ALL of following: key workers/ clinically vulnerable groups (non elderly)/elderly groups 4—Availability for all three plus partial additional availability (select broad groups/ages) 5—Universal availability

**Figure 1:**
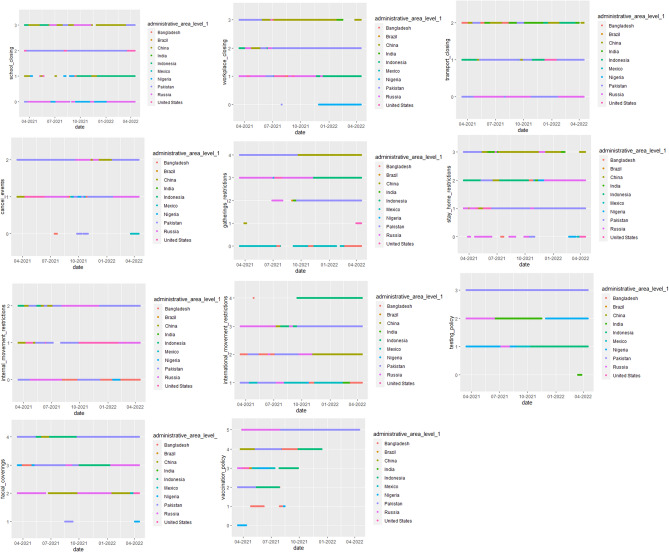
Policies of Countries during COVID-19.

In this study, the main purpose of the study is not to evaluate the policies implemented by the countries in each period or time series. But, all periods are taken into account in the data in general, a change has been observed in the policies of the countries. Therefore, the policies generally applied in the study are to cluster the countries depending on the COVID-19 statistics. In the period, the status of the social policies implemented by ten countries that were the subject of work is shown in detail in the graphs. These 11 social policies, which we call categorical variables, play an important role in the clustering of countries. Therefore, dot plots have been preferred in the visual representation of these variables. Therefore, each of the social policies that change over time has been included in the model as data and their effects have been evaluated. However, no time series analysis has been performed. These changes have been shown in the graphs. The time-varying policies of countries are shown in Fig. 1 (Fig. 1).

### Statistical analysis

The study examined data from the time periods of 15.03.2021 and 15.04.2022. The COVID-19 packages in the R programming were used to collect the data. Calculations were made with dplyr, tidyverse, ggplot2, scales, cluster, fclust, and clustMixType packages in the R program. The relationships between the population and the number of confirmed, tests, and vaccines were examined with the Pearson correlation coefficient. For the variables with a significant relationship, standardization was performed depending on the population of the country with the highest population. The FKM method, which is among the fuzzy cluster algorithms, was used to investigate the clustering of the countries depending on various characteristics over the time period. In order to find the best cluster structure for the method, PC, PE, XB, and SIL.F indexes were used to evaluate the performance of the clusters. As a second clustering method, the K-prototype algorithm was used. In the method, cluster validities were evaluated with the Elbow method. Total within the standard sum of squares (ss) was used for performance comparisons between the two clustering methods. The statistical significance level was taken as 0.05 in the calculations.

## Results

Clustering procedures were conducted for the 10 most populous countries using COVID-19 data between March 15, 2021, and April 15, 2022 (Russia, China, Brazil, United States, India, Pakistan, Indonesia, Bangladesh, Mexico, and Nigeria) by using FKM and K-prototype clustering algorithms in terms of the features described in the material and method part. Descriptive statistics values for all countries according to their COVID-19 status were given in Table 2 (Table 2).

**Table 2:** Distributions of policy situations for all countries between 15.03.2021 and 15.04.2022.

		n	%
School closures	0	646	0.16
	1	679	0.17
	2	1412	0.36
	3	1229	0.31
Workplace closures	0	164	0.04
	1	1112	0.28
	2	1988	0.50
	3	702	0.18
Cancel events	0	65	0.02
	1	1508	0.38
	2	2393	0.60
Gathering restrictions	0	416	0.104
	1	24	0.006
	2	736	0.186
	3	1009	0.254
	4	1781	0.450
Transport closures	0	985	0.25
	1	2280	0.57
	2	701	0.18
Stay home restrictions	0	202	0.05
	1	1774	0.45
	2	1455	0.37
	3	535	0.13
Internal movement restrictions	0	914	0.23
	1	614	0.15
	2	2438	0.62
International movement restrictions	1	835	0.21
	2	1253	0.32
	3	1448	0.37
	4	430	0.10
Testing policy	0	12	0.003
	1	942	0.237
	2	480	0.121
	3	2532	0.639
Facial coverings	1	39	0.010
	2	758	0.191
	3	1514	0.382
	4	1655	0.417
Vaccination policy	0	28	0.007
	1	56	0.014
	2	328	0.083
	3	292	0.074
	4	824	0.208
	5	2438	0.614

Before conducting the clustering analysis, the data was altered by investigating the relationship between the populations of countries and their corresponding confirmed cases, deaths, testing, and vaccines. If a correlation was found between these variables, standardization was carried out based on the population. The study began by exploring the connections between the populations of different countries and their deaths, testing, confirmed deaths, and vaccines. As a result of the review, statistically significant positive correlations were found between the population and confirmed, tests and vaccines (p < 0.05 for each). However, there was no statistically significant relationship between population and death (p = 0.164). The method of standardization was implemented for variables and other countries with respect to the population of China, which is the largest country by population. For example, the standardization was obtained for confirmed cases as,

Standardized Confirmed value of A country = Confirmed value of A country*(China population size/Population size of A country).

To evaluate the policy conditions and standardized variables of all countries, the FKM algorithm was used initially. Different cluster index values were compared to identify the optimal number of clusters for the fuzzy algorithm. Cluster quality was assessed using the PC, PE, SIL.F, and XB cluster indices. The number of clusters was assessed within the range of 2 to 9, and the optimal number was determined. After evaluating the indices, it was found that the ideal number of clusters was 2. Cluster 2 had the highest PC and SIL.F values, and the lowest XB and PE values, making it the best model (Table 3).

**Table 3:** PC, PE, SIL.F, and XB values for different cluster size.

	Cluster index			
Cluster size	PC	PE	SIL.F	XB
2	0.99964	0.00128	0.98794	0.00579
3	0.94575	0.09836	0.83549	0.10535
4	0.94468	0.10085	0.82902	1.19571
5	0.90021	0.17998	0.76875	0.80136
6	0.93743	0.13504	0.79513	0.45379
7	0.93542	0.13852	0.78834	3.13353
8	0.94041	0.13124	0.82136	0.26782
9	0.93202	0.13297	0.85105	0.15570

The FKM clustering results revealed that Brazil, Mexico, Nigeria, Bangladesh, the US, Indonesia, Russia, and Pakistan were grouped into the first cluster across all time intervals. On the other hand, India and China were clustered together in the second cluster, regardless of the time interval being analyzed.

**Figure 2:**
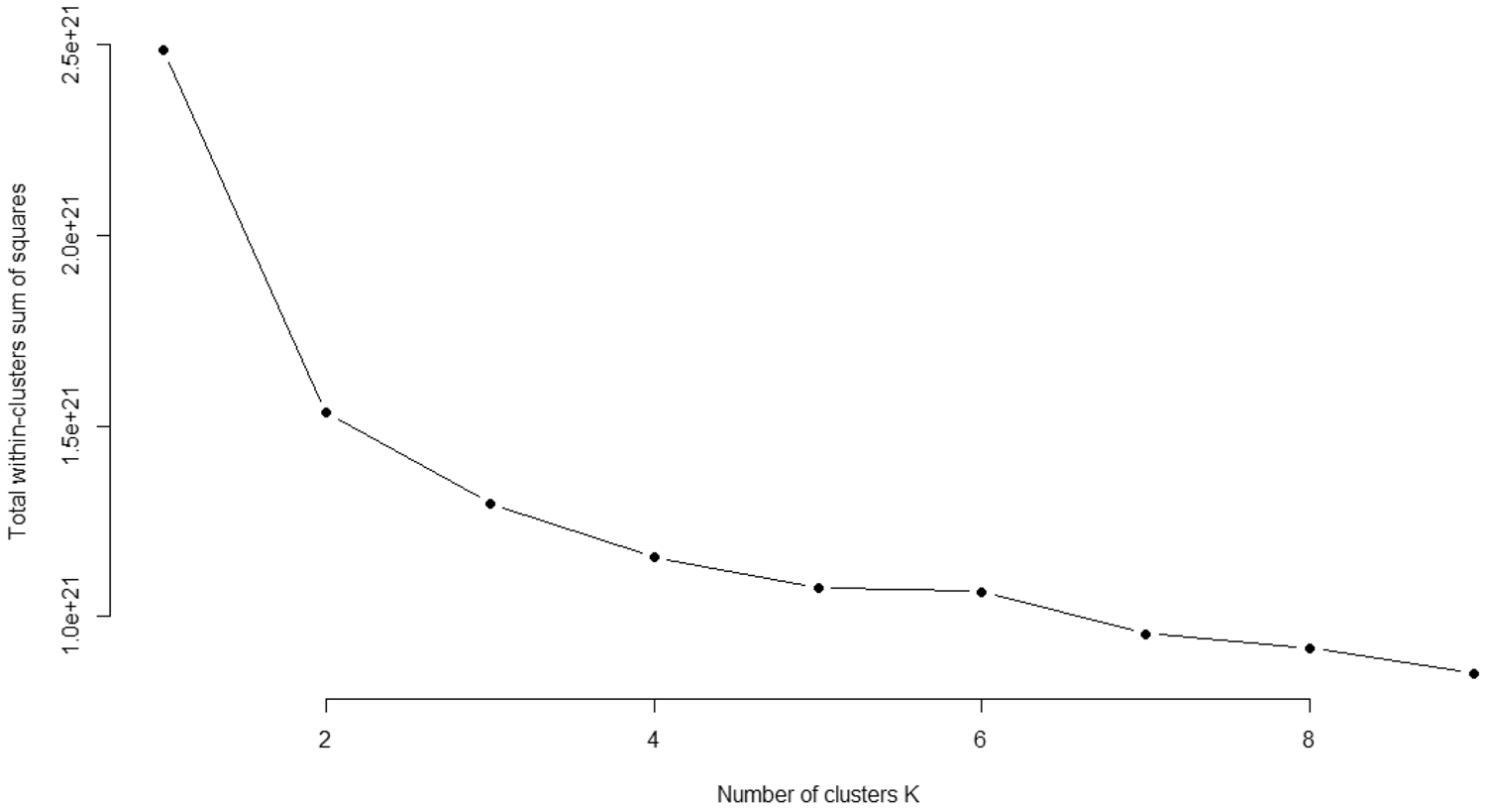
Number of clusters determination with Elbow method.

The K-prototype method was used as another clustering technique, and the appropriate number of clusters was determined using the Elbow method. The ideal number of clusters was identified as the one with the most significant bend in the graph produced by the Elbow technique. The Elbow approach was used to determine a reasonable number of clusters in the line plot for clustering. Based on the graph, the scenario with two clusters had the most significant bend. Therefore, the K-prototype method, similar to the FKM method, was found to have an optimal number of clusters of two (Fig. 2).

After evaluating the two clusters formed by the FKM method, it was found that Brazil, Mexico, Nigeria, Bangladesh, the US, Indonesia, Russia, and Pakistan were clustered into the first group, while India and China formed the second group across all time intervals. It was noted that the cluster memberships of these countries remained constant during the specified time intervals. Clustering is a method used to group entities based on their shared characteristics^14^. Thus, entities belonging to the same cluster exhibit greater similarity in terms of the relevant characteristics compared to entities in other clusters. Conversely, individuals in distinct clusters are composed of samples and individuals with distinct characteristics. The within sum of squares (within ss: Vector of within-cluster sum of squares) statistics indicates how dissimilar the samples are between clusters. It is a metric used to assess how well various clustering techniques function. More related objects are found in a cluster when “within ss” is smaller and vice versa. A good cluster will therefore have small within ss and samples that are highly comparable to one another. We have two values of “within ss”. Because we had two clusters. Thus, the values of within ss were reported in the following. The sums of squares within the cluster were obtained in order to compare the performance of the FKM and K-prototype approaches in cluster separation. The overall inside ss and within ss values acquired by FKM, according to the results, were less than those obtained by the K-prototype technique. This result shows that the FKM method performs better in terms of cluster separation for the data set than the K-prototype method (Table 4).

**Table 4:** FKM and K-prototype sum of squares.

	FKM	Within SS	Total within SS
FKM	Clus1	3.082896e + 19	3.139346e + 19
	Clus2	5.645052e + 17	
K-prototype	Clus1	1.301795e + 21	1.533968e + 21
	Clus2	2.321729e + 20	

Table 5 displays the statistical information for the cluster generated by the FKM method. As FKM is a soft clustering technique, descriptive statistics for membership degrees across clusters are reported. Table 5 shows the descriptive values of membership coefficients in the first and second clusters.

**Table 5:** Descriptive statistics for the membership degrees by clusters.

	Minimum	Q1	Mean	Median	Q3	Maximum
Cluster 1	0.9940443	0.9999754	0.9997743	0.9999876	0.9999921	1.0000000
Cluster 2	0.9999992	0.9999997	0.9999997	0.9999998	0.9999998	0.9999999

Table 6 illustrates the distribution rates of the variables across the two clusters identified using the K-prototype method (Table 6).

**Table 6:** The membership possibilities of variables categories according to K-Prototype.

		*Cluster 1*	*Cluster 2*
School closures	0	0.185	0.074
	1	0.201	0.050
	2	0.383	0.247
	3	0.230	0.628
Workplace closures	0	0.052	0.000
	1	0.344	0.026
	2	0.512	0.457
	3	0.092	0.516
Cancel events	0	0.020	0.000
	1	0.442	0.135
	2	0.538	0.865
Gathering restrictions	0	0.131	0.000
	1	0.005	0.009
	2	0.189	0.173
	3	0.309	0.035
	4	0.365	0.783
Transport closures	0	0.242	0.273
	1	0.673	0.183
	2	0.085	0.544
Stay home restrictions	0	0.064	0.000
	1	0.523	0.145
	2	0.374	0.338
	3	0.039	0.518
Internal movement restrictions	0	0.279	0.035
	1	0.179	0.058
	2	0.542	0.907
International movement restrictions	1	0.244	0.077
	2	0.270	0.497
	3	0.350	0.426
	4	0.136	0.000
Testing policy	0	0.000	0.015
	1	0.297	0.000
	2	0.072	0.317
	3	0.631	0.668
Facial coverings	1	0.012	0.000
	2	0.119	0.479
	3	0.450	0.110
	4	0.419	0.412
Vaccination policy	0	0.009	0.000
	1	0.018	0.000
	2	0.101	0.010
	3	0.087	0.021
	4	0.179	0.324
	5	0.607	0.645

**Figure 3:**
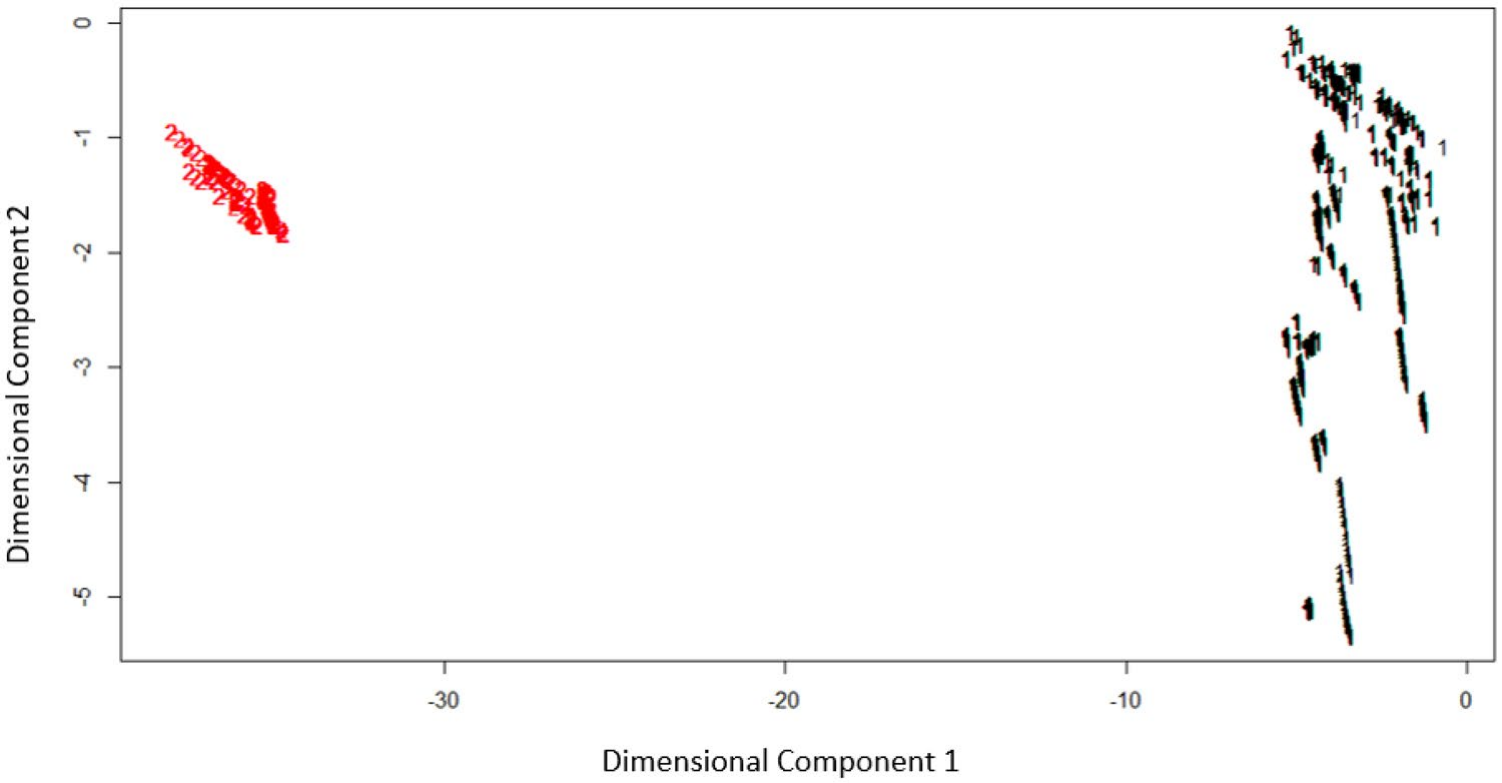
Cluster patterns of Fuzzy K-means and K-prototype.

Similar clustering structures were obtained for both Fuzzy k-means and K-prototype clustering methods used in the study (Fig. 3). For both methods, China and India have separated from other countries and are included in the same cluster for this mixed data structure with multiple variables. Regarding which method is more effective, the within-cluster sum of squares (within.ss) results were examined for both methods to make a judgment.

## Discussion

Since late 2019, the COVID-19 pandemic has rapidly spread across the globe. Medical research has investigated the correlation between COVID-19 and multiple ailments, while various vaccines have been developed. The number of studies on this topic continues to increase in the literature. In response to the COVID-19 outbreak, countries have implemented policies and strategies, which must be managed in accordance with the spread of the disease.

Changing national policies from a specified timeframe and COVID-19 data were assessed in this study, and clustering was performed by incorporating the ten most populated counties in the study. Clustering methods are effective tools for assessing the spread of the pandemic. This study utilized the FKM and K-prototype algorithms among the many clustering techniques.

These algorithms, which are some of the more sophisticated techniques, improve the quality of our research.

No existing literature has combined the methods used in this study. The study aimed to evaluate the relationship between COVID-19 numerical data and population size and standardized the data according to the population. Significant correlations were observed and confirmed, leading to tests being conducted, vaccine variables being determined, and the population size effect being eliminated by standardization. Cluster performance was evaluated using various criteria, including the sum-of-squares, which showed that the FKM method performed better than the K-prototype method. The study resulted in two clusters: the first composed of Brazil, Mexico, Nigeria, Bangladesh, the United States, Indonesia, Russia, and Pakistan, and the second composed of India and China. These clusters remained consistent throughout the specified time frame.

Mahmoudi et al. published a study in 2020, where they used the fuzzy clustering method to cluster selected countries such as the United States, Spain, Italy, Germany, the United Kingdom, France, and Iran. They used data from February 22, 2020, to April 2020 and investigated the relationship between population size and the spread of COVID-19, similarly to our study. They standardized the data based on population size before conducting the cluster analysis, considering confirmed cases, dead cases, cumulative confirmed cases, and cumulative dead cases. Fuzzy clustering showed that Italy and Spain differed from other countries^15^.

Afzal et al. published a study in 2021, where they used the c-means and fuzzy c-means clustering algorithms with COVID-19 data. They determined the optimal number of clusters using various indices, including PC, XB, the Fukuyama-Sugeno index, the validity function, Zahid separation compaction, and entropy indices. The study assessed countries' longitude, latitude, date, reported cases, recovered, and death properties while applying the clustering algorithms. The results showed that the fuzzy c-means (k-means) clustering algorithm provided better results than the c-means algorithm^16^.

D'Urso et al. published a study in 2021, that involved applying the spatial fuzzy clustering algorithm to COVID-19 data for 20 Italian regions. They used the exponential distance-based fuzzy c-medoids method based on the B-spline for time-series clustering, considering geographical locations in the process. The study aimed to cluster countries for various purposes. D'Urso et al. identified three distinct clusters of 20 Italian regions in their study^17^.

Liu et al. proposed a new approach for optimizing cluster centers in their work published in 2019. The research aimed to enhance the clustering performance of K-Means, which is the most widely used hard clustering method. They developed a clustering technique based on randomly occurring distributedly delayed PSO (RODDPSO) and compared its effectiveness with the mean silhouette values of the K-means and FCM clustering algorithms. In contrast, our study focuses on the variable structure of our COVID-19 dataset, specifically clustering a mixed data set made up of both categorical and numerical observations. Therefore, we utilize the FCM algorithm, which is frequently used in numerical data and is the most well-known approach among soft clustering methods, and the K-Prototype method, which is preferred only for clustering mixed data. Additionally, we compare the performances of the clustering methods for mixed data structures, while Liu et al. aimed to increase the performance of the hard clustering algorithm used in their suggested approach, utilizing intra-cluster sum-of-squares statistics in the process^18^.

In their 2020 study, Liu et al. applied a deep belief network (DBN) to the RODDPSO-based clustering algorithm that they previously suggested to classify patients who visited an emergency department of a hospital in London. This highlights the potential for further research to be conducted by incorporating other classification algorithms with the clustering methods used in our study, and revisiting the clustering of our COVID-19 mixed data set based on countries^19^.

Li et al. proposed a new method called the ranking system-based Particle Swarm Optimization (RSPSO) with dynamic learning strategies for complex optimization problems in their 2019 and 2020 studies, which is similar to the studies conducted by Liu et al. However, it should be noted that our study does not address any optimization problems. Furthermore, it is worth noting that the FKM and K-Prototype algorithms used in our study and the approach employed by Li et al. in PSO share a similar general logic^20^. Li et al. utilized PSO as a clustering algorithm in their study, where each particle in the swarm represents a possible solution for the clustering problem. In this approach, the particles represent cluster centers, and they aim to find the best centers for the clusters. The data points are assigned to the nearest cluster based on their distance from these centers, and the optimal clustering solution is found. In our study, we used the Fuzzy C-Means and K-Prototype clustering algorithms, which operate on a similar principle. These clustering algorithms are appropriate for our mixed data set structure. Furthermore, we conducted statistical evaluations to compare the clustering performances of these two methods in our study.

This study differs from previous studies in that it uses a wide time range of 15.03.2021 to 15.04.2022 and evaluates the top ten countries with the highest population densities. The study evaluates the relationship between population size and COVID-19 data, which has been neglected in previous studies. The clustering process considers a mixed data structure and evaluates policies adopted by countries, including school and transportation closures, along with COVID-19 data. The study uses two clustering algorithms, FKM and K-prototype, to obtain optimum clusters by evaluating different clustering indices, and cluster quality tests are performed to select the number of clusters for FKM and K-prototype. The statistical techniques used in this study make it unique and innovative.

In summary, our study is expected to make a valuable contribution to the literature by utilizing advanced clustering algorithms to categorize the population data of ten countries with large populations, determining the optimal number of clusters using appropriate statistical methods. By using these clusters generated by the advanced statistical methods, it is possible to analyze the health policies of different countries. However, it would be more comprehensive if the study could include countries from all around the world.

## Data Availability

The datasets generated and analyzed during the current study are available in the R package. [https://cran.r-project.org/web/packages/COVID19/index.html].
